# The Tela Cutis Nasi Flap: A Technical Note on Nasal Sill Reconstruction in Secondary Cleft Rhinoplasty

**DOI:** 10.3390/jcm15083139

**Published:** 2026-04-20

**Authors:** Łukasz Banasiak, Oskar Komisarek, Vanessa Olichwer, Paweł Radkowski, Paweł Burduk, Krzysztof Dowgierd

**Affiliations:** 1 Department of Plastic Surgery, Regional Specialist Hospital in Olsztyn, 10-228 Olsztyn, Poland; 2 Center for Craniofacial Anomalies, Voivodeship Children’s Hospital in Olsztyn, 10-561 Olsztyn, Poland; 3 Department of Otolaryngology, Audiology and Phoniatrics, Collegium Medicum, Nicolaus Copernicus University in Toruń, 85-067 Bydgoszcz, Poland; 4 Students Scientific Association of Maxillofacial Surgery, Faculty of Medicine, Collegium Medicum, Nicolaus Copernicus University in Toruń, 85-067 Bydgoszcz, Poland

**Keywords:** cleft rhinoplasty, nasal sill, local flap, secondary cleft surgery, nostril base, fibro-adipose flap, technical note, surgical technique

## Abstract

**Background:** Despite advances in cleft rhinoplasty, the nasal sill remains an underappreciated subunit, yet it plays a crucial role in nostril symmetry and aesthetic balance. Hypoplasia or absence of the nasal sill on the cleft side often persists after primary repair and may complicate secondary nasal base correction. Current methods for sill reconstruction are limited by donor site morbidity, variability in tissue match, or non-anatomic tissue substitution. **Methods:** This technical note describes a surgical protocol utilizing the Tela Cutis Nasi flap, a pedicled fibro-adipose flap harvested from the adjacent nasal base, to reconstruct the deficient sill in patients with previously repaired unilateral cleft lip. The flap concept, anatomical rationale, stepwise operative steps, and patient selection considerations are outlined. **Results:** This technical note details the surgical steps, anatomical rationale, and flap design. No formal morphometric or patient-reported outcome analysis is included in this report; these data are being collected within an ongoing prospective outcome study designed to evaluate efficacy and long-term stability. **Conclusions:** The Tela Cutis Nasi flap is intended as an anatomically based local option for nasal sill reconstruction that can be integrated into secondary cleft nasal base surgery. This article contributes a standardized operative description, indications, technical constraints, and anticipated pitfalls, without assessment of clinical outcomes or long-term stability.

## 1. Introduction

Cleft lip and palate (CLP) is among the most common congenital craniofacial anomalies and frequently results in a complex, three-dimensional deformity of the nose. Even after primary repair, many patients present with persistent nasal asymmetry that motivates secondary cleft rhinoplasty during adolescence or adulthood [1,2]. The deformity typically involves alar base malposition, columellar shortening, tip deviation, and collapse of the external nasal valve and has been extensively discussed in the literature [3,4,5].

Within this spectrum, the nasal sill represents a small but functionally and aesthetically important subunit. It contributes to nostril shape, base symmetry, and the transition between the columella and the upper lip [6]. On the cleft side, the sill is frequently hypoplastic, flattened, or absent, which may persist despite well-performed primary lip repair. A deficient sill can accentuate nostril base asymmetry and may complicate secondary rhinoplasty planning. Nevertheless, in many standard descriptions of cleft nasal correction, the sill is mentioned only briefly or addressed indirectly through alar base repositioning rather than by a dedicated reconstructive maneuver [4,7].

Several approaches have been proposed to restore the nasal sill in cleft patients, including local skin and muscle rearrangements at the alar base, nasolabial flaps, composite tissue grafts, and structural augmentation with cartilage or alloplastic materials [3,8]. While such techniques can modify nostril base shape, they may be associated with additional visible scars, donor-site morbidity, or the use of non-anatomic tissue substitutes. In addition, many methods focus on skin and muscle repositioning, with less emphasis on reconstituting the subcutaneous fibro-adipose volume that normally supports the sill.

In clinical practice, there remains a need for a simple, anatomically based technique that specifically targets the cleft-side nasal sill using local tissue, with minimal additional scarring and without extending the external rhinoplasty incision pattern. Ideally, such a method would provide autologous volume beneath the sill and allow precise three-dimensional positioning at the nostril base within the broader framework of secondary cleft rhinoplasty.

In this technical note, we describe the Tela Cutis Nasi flap, a pedicled fibro-adipose flap harvested from the nasal base and rotated into the cleft-side sill region. The aim is to offer a standardized technical description for sill reconstruction that may complement existing cleft rhinoplasty techniques. This manuscript provides a technical protocol only and does not assess clinical outcomes or long-term stability.

The technique presented in this study introduces a standardized approach to nasal sill reconstruction based on the transfer of a pedicled fibro-adipose flap from the adjacent nasal base. Unlike previously described methods that rely on skin, muscle, or composite grafts, the Tela Cutis Nasi flap specifically targets the subcutaneous fibro-adipose layer corresponding to the native sill tissue. The flap is designed with a defined pedicle, pivot point, and arc of rotation, allowing controlled three-dimensional positioning within the nasal base without the need for additional external incisions or distant donor sites. This anatomically oriented concept aims to provide a reproducible and tissue-specific solution for selected cases of nasal sill deficiency in appropriately selected patients. 

## 2. Scope, Setting, Ethics, and Consent

This manuscript presents a structured surgical technique protocol detailing a secondary reconstructive approach for the nasal sill in patients with unilateral cleft lip, with or without cleft palate. The primary objective is to provide a standardized description of the Tela Cutis Nasi flap procedure, including operative steps and criteria for patient selection.

The described procedure reflects an evolution of routine secondary cleft rhinoplasty practice and does not modify the accepted standard of care. The work was conducted in accordance with the principles of the Declaration of Helsinki. According to national regulations and institutional policies, technical descriptions that do not report identifiable patient data and do not involve deviation from standard care are exempt from formal institutional review board approval.

All procedures were performed at the Department of Plastic Surgery, Regional Specialist Hospital in Olsztyn, Poland, in collaboration with the Center for Craniofacial Anomalies at the Voivodeship Children’s Hospital in Olsztyn, by a single senior surgeon experienced in cleft lip and nasal base reconstruction.

If intraoperative images are included, they are presented in anonymized form without identifiable features. Written consent for publication of anonymized images was obtained where applicable.

Artificial intelligence (AI) tools were used to support language editing and improve the clarity of the manuscript. The authors reviewed and validated all outputs generated by the AI tool and take full responsibility for the content of the publication.

Definition of the Tela Cutis Nasi Flap

The Tela Cutis Nasi flap refers to a pedicled fibro-adipose (subcutaneous) tissue flap harvested from the nasal base adjacent to the alar–facial junction. The term is used as a descriptive designation introduced to standardize the anatomical layer and flap concept for international readership.

Indications and Contraindications

Indications

Candidates are eligible for the Tela Cutis Nasi flap procedure if they meet all of the following criteria:

- Unilateral cleft lip (UCL) or unilateral cleft lip and palate (UCLP) with completed primary repair.
- Presence of cleft-side nostril base asymmetry characterized by a flattened, hypoplastic, or poorly defined nostril sill.
- Availability of adequate subcutaneous tissue at the nasal base to permit flap elevation, confirmed by clinical and intraoperative assessment.

Morphological Classification of Nasal Sill Deformity

For practical surgical decision-making, nasal sill deformities can be categorized based on their morphological presentation. In this study, a simplified classification is adopted:

- Type I—flattened nasal sill with preserved but reduced contour
- Type II—hypoplastic nasal sill with insufficient subcutaneous volume
- Type III—absent or severely deficient nasal sill

The Tela Cutis Nasi flap is primarily intended for patients presenting with Type I and Type II deformities, where local fibro-adipose tissue is available for reconstruction. This simplified framework is consistent with previously proposed classification systems, including the morphology-based classification described by Xia et al. [7], which links nasal sill form to tailored surgical strategies.

Contraindications and caution

Patients are excluded from the Tela Cutis Nasi flap procedure if any of the following are present:

- Bilateral cleft lip deformities requiring different reconstructive strategies.
- Severe nasal deformities necessitating comprehensive open septorhinoplasty (e.g., pronounced septal deviation, tip ptosis, or dorsal collapse).
- History of prior aggressive surgical manipulation at the cleft-side sill, such as full-thickness wedge resections or extensive scarring that would preclude local flap elevation.
- Any contraindication to elective surgery or anesthesia, including uncontrolled systemic illness or active infection.

Use in revision cases:

The technique can be considered in revision settings; feasibility depends on scar pattern and pedicle integrity and requires individualized intraoperative assessment.

Surgical Protocol

**Figure 1:**
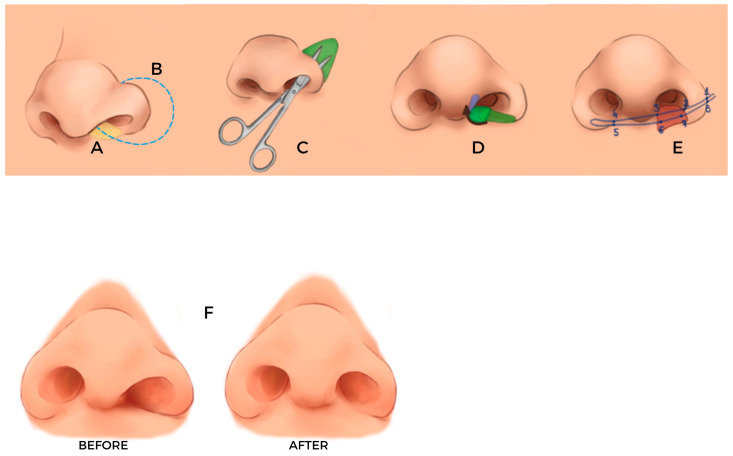
Schematic overview of the surgical technique. Illustrated step-by-step sequence including: (**A**) anatomical marking of the nasal base and planned sill position; (**B**) incision design at the upper lip/nasal sill junction; (**C**) elevation of the pedicled fibro-adipose Tela Cutis Nasi flap with identification of the pedicle; (**D**) flap rotation around the defined pivot point, demonstrating the arc of rotation toward the nasal sill pocket; (**E**) flap inset and fixation within the prepared sill pocket; (**F**) final intraoperative configuration after flap inset and closure.

The Tela Cutis Nasi Flap procedure is performed under general anesthesia and may be conducted as a standalone intervention or in conjunction with other secondary cleft lip/nasal revisions. The following operative steps describe the standardized technique as applied in eligible patients. A schematic step-by-step overview of the procedure is presented in Figure 1.

### 2.1. Preoperative Marking and Anesthesia

**Figure 2:**
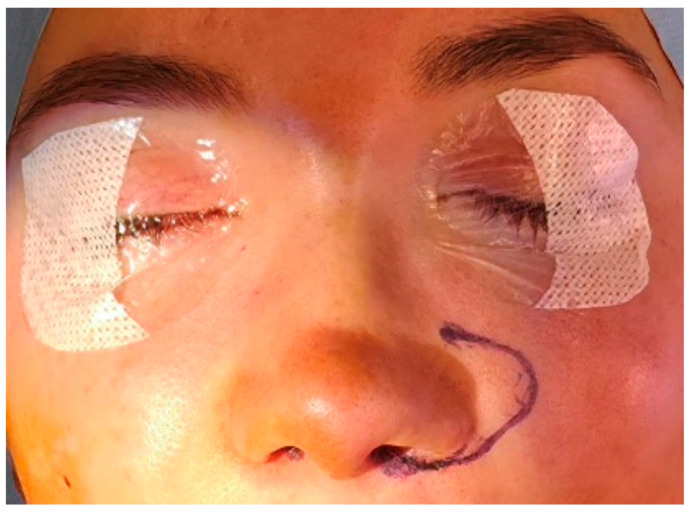
Preoperative marking of anatomical landmarks. The alar–facial junction, columellar base, and intended position of the reconstructed nasal sill on the cleft side are marked with the patient in the supine position. Symmetry with the non-cleft nostril is used as the primary reference.

With the patient in a supine position, key anatomical landmarks are identified and marked, including the alar–facial junction and columellar base (Figure 2). The planned position of the reconstructed nostril sill is outlined on the cleft side, aiming for symmetry with the contralateral nostril. Local infiltration with a vasoconstrictive anesthetic solution (e.g., lidocaine with epinephrine) is administered at the nasal base and upper lip to provide hydrodissection and hemostasis.

### 2.2. Incision and Flap Design

A small curvilinear incision (approximately 5–8 mm) is made at the junction of the upper lip and the nasal sill, extending from near the columella toward the pyriform aperture. Because the incision lies entirely within this natural transition zone, the resulting scar is usually short and inconspicuous. Through this access, a portion of the Tela Cutis Nasi flap is first de-epithelialized and the flap is then elevated from the subcutaneous tissue layer. This flap consists of fibrofatty tissue, potentially incorporating fibers from the orbicularis oris or nasalis muscles. The flap is pedicled medially near the columellar base, which allows rotation toward the nasal sill while maintaining vascular supply. This orientation enables controlled superomedial transposition of the flap without the need for pedicle division.

### 2.3. Flap Elevation and Mobilization

Sharp dissection is used to elevate the flap in a subcutaneous plane superficial to the mimetic musculature of the upper lip, taking care to preserve the fibro-adipose tissue and its perforating vascular supply. The flap is raised from caudal to cranial with the pedicle located at the superior aspect of the incision, where branches of the superior labial artery are presumed to contribute to perfusion based on regional anatomy, without formal vascular mapping. Care is taken to avoid excessive thinning or skeletonization of the pedicle. The dissection is extended medially toward the columellar base and laterally toward the alar base as required to obtain sufficient length and mobility while maintaining a broad attachment to the nasal base.

**Figure 3:**
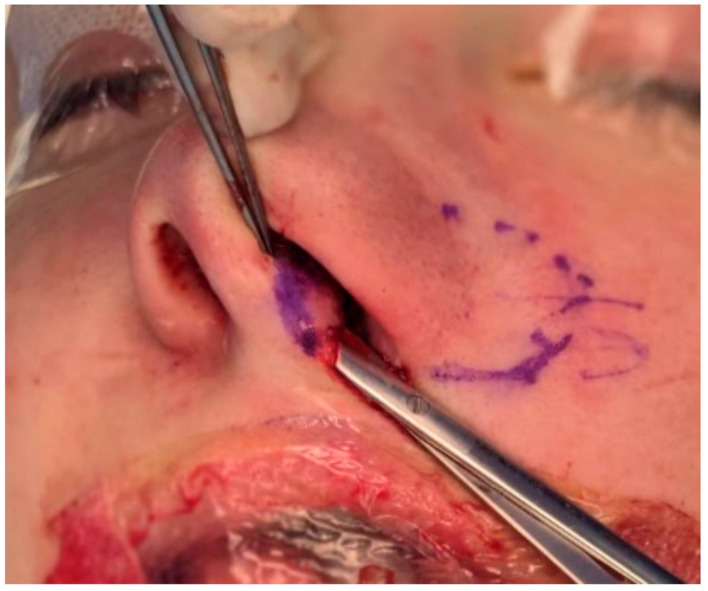
Elevation of the Tela Cutis Nasi flap. The fibro-adipose flap is carefully elevated in a subcutaneous plane, with preservation of the medial pedicle near the columella.

After flap elevation, a subcutaneous tunnel is created toward the planned sill pocket and, when indicated, toward the columella. Depending on the planned inset, the cutaneous portion of the flap may be partially de-epithelialized to facilitate its passage through a subcutaneous tunnel and to prevent epithelial entrapment beneath the skin. The remaining epithelialized portion is preserved where surface lining or contour definition of the nasal sill is required (Figure 3).

### 2.4. Flap Inset and Fixation

**Figure 4:**
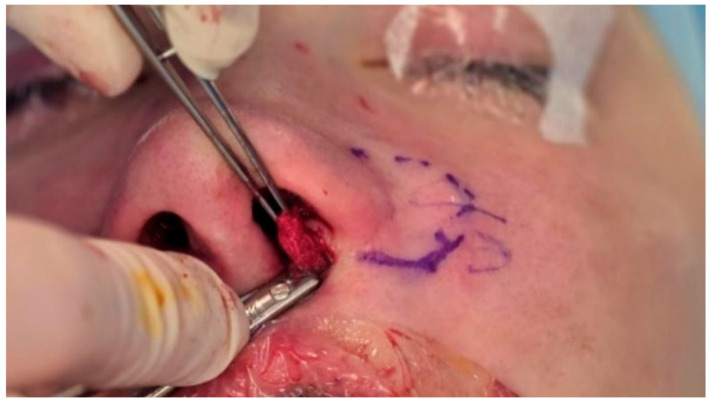
Flap rotation and positioning. The flap is rotated superomedially and advanced into the cleft-side nostril sill region.

**Figure 5:**
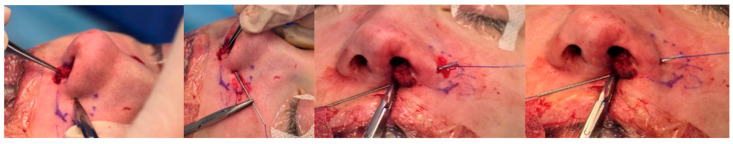
Flap fixation using deep sutures. The flap is anchored to the periosteum of the anterior nasal spine or pyriform aperture using absorbable sutures. Additional sutures approximate the flap to surrounding tissues.

The flap is positioned to augment the sill subunit, occupying the space between the columella and the alar base on the cleft side (Figure 4). It is rotated superomedially into the prepared pocket so that the cutaneous portion is positioned to re-establish continuity of the nostril sill, while the de-epithelialized portion provides underlying volume and support. A transflap suture is then passed through the subcutaneous portion of the flap to control the vector of rotation and advancement; this suture is anchored to periosteal structures such as the anterior nasal spine or the ipsilateral pyriform rim, depending on the direction of the required base support (Figure 5). This maneuver is intended to stabilize the flap in three dimensions and to approximate basal widening of the columella at the cleft-side nostril base. Additional absorbable sutures are placed to integrate the flap with the surrounding soft tissues and to smooth the sill contour. If an external incision has been used, it is closed in layers with fine sutures, aligning the skin edges precisely within the upper lip/nasal sill junction to minimize scar visibility.

### 2.5. Adjunctive Procedures

Depending on the individual deformity, supplementary maneuvers such as cartilage grafting (e.g., rim or columellar strut grafts), alar base repositioning, tip refinement, and limited septal correction may be performed in conjunction with the Tela Cutis Nasi flap. These adjunctive procedures can be carried out either through the same upper lip/nasal sill junction incision, through standard lip or vestibular approaches, or as part of an open rhinoplasty exposure when a more extensive correction of the nasal framework is required. In our clinical practice, the Tela Cutis Nasi flap is typically incorporated into broader secondary cleft rhinoplasty rather than used in isolation. The presence of the flap does not preclude or substantially alter these adjunctive maneuvers, but instead is integrated into such combined procedures without changing the overall surgical plan.

### 2.6. Closure and Postoperative Care

Upon completion of flap inset and soft-tissue rearrangement, the external incision is closed in layers. The deep dermis is approximated with fine absorbable sutures (e.g., 5-0 polyglactin), and the skin is closed with interrupted non-absorbable sutures (e.g., 6-0 polypropylene), taking care to align the wound precisely within the upper lip/nasal sill junction to minimize scar visibility. Non-absorbable skin sutures are typically removed between postoperative days 5 and 7. A light adhesive dressing or nasal taping is routinely applied across the nasal base and upper lip to support the reconstructed sill and to limit early edema; this is usually maintained for approximately 7–10 days. Nasal stents or intranasal splints are not routinely required solely for the sill reconstruction, but may be used when dictated by concomitant septal or tip work.

Patients are advised to avoid nose blowing, manipulation of the nasal base, and contact sports for at least 4–6 weeks after surgery. Most visible edema at the sill and columellar base decreases substantially within 2–3 weeks, although mild induration or subtle contour changes may continue to improve over several months. Standard follow-up visits are scheduled in the early postoperative period (e.g., at 1 week and 1 month) to monitor wound healing, with subsequent assessments coordinated with the broader cleft rhinoplasty follow-up protocol to document wound healing and tissue integration.

## 3. Discussion

Secondary correction of the unilateral cleft nasal deformity requires an individualized, multi-component approach that addresses both cartilaginous framework abnormalities and soft-tissue deficiencies. While alar cartilage repositioning and nasal tip refinement remain central elements of secondary cleft rhinoplasty, the nasal sill—an essential subunit of the nasal base—has historically received less focused attention [8]. Nevertheless, the nasal sill contributes to nostril shape, base width, and the transition between the columella and the upper lip. Hypoplasia or absence of this structure on the cleft side may accentuate nostril base asymmetry and complicate overall nasal base reconstruction, even when cartilaginous correction is appropriately performed [3,7].

In contrast to previously described techniques, the Tela Cutis Nasi flap emphasizes the use of subcutaneous fibro-adipose tissue rather than skin, muscle, or composite grafts. This approach allows reconstruction of the nasal sill using tissue that closely resembles the native structural composition of this subunit. Furthermore, the technique is defined by a consistent pedicle location, pivot point, and arc of rotation, which may facilitate reproducibility and integration into established secondary cleft rhinoplasty procedures. Rather than introducing a completely new reconstructive principle, this method represents a refinement and standardization of local tissue-based reconstruction tailored specifically to the nasal sill. This distinction may be particularly relevant in cases where restoration of subcutaneous volume, rather than surface contour alone, is required.

The Tela Cutis Nasi flap described in this manuscript is designed to address nasal sill deficiency through the transfer of a pedicled fibro-adipose flap harvested from the adjacent nasal base. This approach follows the reconstructive principle of replacing “like with like,” utilizing tissue that is comparable in composition to the native sill region. By maintaining a pedicled vascular supply, the technique is intended to preserve local perfusion while avoiding the need for distant donor sites commonly associated with cartilage or composite grafts [3,5]. The flap concept emphasizes anatomical positioning and integration into standard secondary cleft nasal base procedures rather than standalone sill reconstruction.

Several previously described techniques have highlighted the value of local tissue rearrangement for nasal sill and nasal floor correction. Agarwal et al. reported the use of an orbicularis oris muscle turnover flap to augment the nasal sill without additional external incisions [4]. Rahpeyma et al. proposed a subcutaneous nasolabial flap accessed intraorally to elevate the depressed nasal floor in adult cleft rhinoplasty [3,6]. While conceptually related, the Tela Cutis Nasi flap is presented as a standardized, anatomically targeted method focused specifically on sill reconstruction using a defined pedicle, pivot point, and arc of rotation that can be incorporated into broader secondary rhinoplasty strategies.

As with other local flap techniques, successful execution of the Tela Cutis Nasi flap requires familiarity with cleft nasal base anatomy and careful intraoperative judgment. Dissection is performed in a region that may be affected by prior surgery and scarring, making preservation of the flap pedicle and avoidance of excessive thinning particularly important. Accurate flap design, controlled rotation, and tension-free inset are emphasized to minimize technical pitfalls and to allow appropriate three-dimensional positioning at the nostril base.

Several limitations should be acknowledged. The availability and mobility of local fibro-adipose tissue may vary depending on prior surgical interventions, scar burden, and individual anatomy. In cases with extensive scarring or limited tissue volume, the flap alone may not be sufficient to address all aspects of nasal base deficiency. Although patients requiring comprehensive septorhinoplasty are outside the primary scope of this protocol, adjunctive procedures—such as alar rim or columellar support grafts—may be considered based on intraoperative findings [3,5]. In such settings, the flap is intended to be combined with other reconstructive maneuvers rather than to replace them.

Potential technical risks include compromise of the pedicle if excessively narrowed or tensioned, contour irregularity related to flap bulk or pocket design, tethering along the upper lip/nasal sill junction, secondary asymmetry associated with scar maturation, and malposition of the flap within the sill pocket. These considerations underscore the importance of meticulous surgical technique and individualized planning, particularly in revision cases.

Assessment of nasal base contour behavior, scar maturation, and long-term tissue stability typically requires follow-up of at least 9–12 months using standardized morphometric and patient-reported outcome measures. The present manuscript does not include clinical outcome assessment or long-term follow-up data and is intentionally limited to a technical description of flap design, operative steps, indications, and anticipated pitfalls. Outcome-oriented evaluation of this technique is intended to be reported separately in a dedicated clinical series.

Beyond secondary cleft rhinoplasty, the conceptual principles of the Tela Cutis Nasi flap may warrant consideration in other reconstructive contexts. Xia et al. emphasized the relevance of nasal sill morphology for surgical planning through their proposed classification system [7], reinforcing the need for techniques that specifically target this subunit. Whether similar pedicled approaches could be adapted for use during primary cleft lip repair remains a subject for future investigation and would require careful consideration of growth-related factors [6].

In summary, this technical note aims to provide a standardized and anatomically oriented description of the Tela Cutis Nasi flap as a local option for nasal sill reconstruction. By clearly defining flap design, pedicle orientation, and operative steps, the manuscript establishes a technical framework that may support future validation and outcome-focused studies. This manuscript is intentionally limited to a technical description and does not include clinical outcome analysis or long-term validation, which will be addressed in future studies. At present, the technique should be considered a targeted adjunct within secondary cleft rhinoplasty rather than a standalone reconstructive solution.

## 4. Conclusions

The Tela Cutis Nasi flap represents a standardized local fibro-adipose flap for targeted nasal sill reconstruction in secondary cleft rhinoplasty. Further clinical studies are required to evaluate long-term outcomes and to validate its role within comprehensive nasal base reconstruction strategies.

## Data Availability

The data presented in this study are available on request from the corresponding author (O.K.). All data supporting the findings of this study are included within the article.

## References

[1] Frederick R.M., Dougherty W., Dobratz E. (2025). Secondary columellar lengthening in bilateral nasal cleft deformities with a sliding flap cheilorhinoplasty. Otolaryngol.–Head Neck Surg..

[2] Hassan A.K.A., Hifny M.A., Ali A.A.A., Saied S. (2023). Cleft nasal deformity and rhinoplasty: Review article. J. Pharm. Negat. Results.

[3] Adham G., Keyhan S.O., Fallahi H.R., Ziaei H., Thomas M. (2021). Nasal sill augmentation: An overlooked concept in rhinoplasty—A technical note and review of the literatures. Maxillofac. Plast. Reconstr. Surg..

[4] Park Y.W., Kwon K.J., Kim M.K. (2015). Double-layered reconstruction of the nasal floor in complete cleft deformity of the primary palate using superfluous lip tissue. Maxillofac. Plast. Reconstr. Surg..

[5] Ayhan M., Gorgu M., Erdogan B., Tuncer S. (2006). Various applications of chondrocutaneous composite grafts in secondary cleft lip nose patients. J. Craniofacial Surg..

[6] Rahpeyma A., Khajehahmadi S., Siraji A.T. (2015). Subcutaneous nasolabial flap for eliminating depressed nasal floor in adult cleft rhinoplasty: Technical note. Plast. Reconstr. Surg.–Glob. Open.

[7] Xia Y., Yuan J., Wang Z., Zhen Y., An Y. (2024). A novel classification of nasal sill morphology provides strategies for secondary cleft rhinoplasty. Aesthetic Plast. Surg..

[8] Sykes J.M., Jang Y.J. (2009). Cleft Lip Rhinoplasty. Facial Plast. Surg. Clin. N. Am..

